# Isolated Soft Tissue Cysticercosis Involving the Trunk in Children: Report of 4 Cases

**Published:** 2013-09-24

**Authors:** Shalini Sinha, Abhishek Tiwari, Yogesh Kumar Sarin, Nita Khurana

**Affiliations:** Dept. of Pediatric Surgery, Maulana Azad Medical College, New Delhi, India.; Dept. of Pediatric Surgery, Maulana Azad Medical College, New Delhi, India.; Dept. of Pediatric Surgery, Maulana Azad Medical College, New Delhi, India.; Dept. of Pathology, Maulana Azad Medical College, New Delhi, India.

**Keywords:** Cysticercosis, Taenia solium, Parasitic cyst

## Abstract

Isolated soft tissue cysticercosis of the trunk in the absence of concurrent central nervous system involvement is uncommon and may be difficult to diagnose. We report 4 such cases in the pediatric age group. Preoperative diagnosis of soft tissue cysticercosis was considered only in 1 patient, the rest were diagnosed only after biopsy. Complete excision (without rupture) was done. All of them underwent a CT scan head along with ophthalmic examination to rule out the more common sites of occurrence of cysticercosis. No further treatment was undertaken as the evaluation was negative. In endemic areas like ours we must suspect this entity not only in the limb muscles, but also in the subcutaneous tissues of the trunk. If diagnosed accurately, it can be treated medically, eliminating the need for surgery.

## INTRODUCTION

Human cysticercosis is caused by infestation with larvae of pork tapeworm Taenia solium and is endemic in India.[1] The central nervous system is involved in 60-90% of cases.[2] Soft tissue cysticercosis usually involves skeletal muscles of extremities and less frequently the subcutaneous plane.[3] Isolated soft tissue cysticercosis without central nervous system involvement is uncommon and may be difficult to diagnose.[4] A literature search revealed paucity of data on soft tissue cysticercosis in the pediatric age group. We report four such children who underwent surgical excision of isolated soft tissue cysticercosis involving the trunk (histopathologically proven) over a period of 2 years.

## CASE REPORT

**Case 1**

A 5-year-old boy presented with a painless swelling on the right chest wall, just beneath the sterno-clavicular joint, of 5 months duration. There was no history of preceding trauma. He was a vegetarian residing in an urban locality. On examination there was a spherical, freely mobile swelling, measuring 2 cms in diameter, in the subcutaneous plane. Fine needle aspiration cytology (FNAC) was inconclusive. He underwent excision biopsy with a clinical diagnosis of epidermoid cyst. Histopathology (HPE) however confirmed cysticercus cellulosae surrounded by palisading histiocytic reaction (Fig 1a and b) and inflammation extending into the surrounding musculature along with fibrosis. He did not have any neurological symptoms, seizures or visual disturbances. CT scan head and ophthalmic examination were were unremarkable. Hence no further treatment was undertaken.

**Figure:**
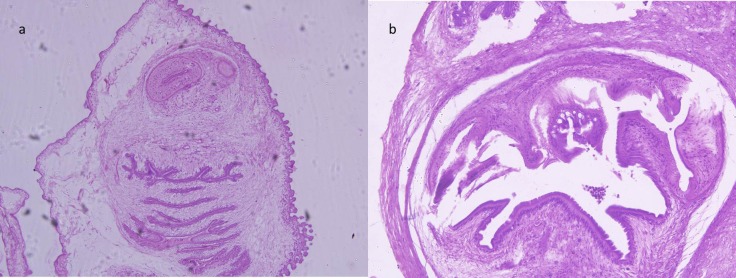
Figure 1: Photomicrograph showing cysticercus larval body stained by a) Hematoxylin and Eosin (x 40) and b) High power view Hematoxylin and Eosin (x 200)

**Case 2**

A 5-year-old boy was brought with a 2 cm swelling in the chest wall noticed for the last 6 months. He was also vegetarian, living near a milk dairy in the city. Clinical examination revealed a spherical, smooth, mobile and firm swelling arising from the subcutaneous tissues of the left 8th intercostal space in the mid-clavicular line. FNAC showed acellular eosinophillic material without any fragments of cyst wall, scolices or hooks. There were no neurological or ophthalmic abnormalities. Evaluation for cerebral and ocular disease was negative. Excision biopsy confirmed the clinical suspicion of parasitic cyst.

**Case 3**

A 4-year-old-boy complained of a painless swelling on his anterior abdominal wall for 7 days. He ate a non-vegetarian diet and belonged to a suburban locality. Clinical findings included a non-tender, spherical swelling, 1.5 cm in diameter, in the right lumbar region close to anterior axillary line.It was arising from the subcutaneous plane and was freely mobile. Hence neither sonographic evaluation nor FNAC were done. With a clinical impression of lipoma,he was taken up for surgical excision, which revealed cysticercus cellulosae with surrounding inflammation. No further treatment was undertaken as the neuro-ophthalmic evaluation was negative.

**Case 4**

A 12-year-old girl noticed an abdominal wall swelling for the last one month. It was insidious in onset, painless and non-inflammatory. She was a vegetarian from the suburban area. There were no similar swellings elsewhere in the body. There was a 1.5 x 1.5cm, firm, spherical swelling in the subcutaneous tissues of the left paraumbilical region. The lesion was too small for FNAC or ultrasonography. Clinically thought to be a neurofibroma, the cyst removed at surgery was found to be cysticercosis. Further investigations did not reveal any foci of cysticercosis in the brain or eye.

## DISCUSSION

The incidence of cysticercosis in India is alarmingly high though the exact figures are not known.[1] In a study done in rural India, the sero-prevalence of cysticercosis was found to be 22.4% and was known to increase with age.[5] Humans become dead-end hosts of the T. solium larvae when they drink contaminated water or eat raw/ poorly cooked vegetables or pork infested with larvae.[2] As was seen in our patients, cysticercosis is equally common in vegetarians. The reason being lack of basic sanitation facilities that promote faecal contamination of improperly washed uncooked food.[1]

The most common site of occurrence of soft tissue cysticercosis is skeletal muscles of the upper extremities.[3] Abdominal and chest wall lesions are seen less often.[6,7] The subcutaneous nodules are usually painless. However, rupture of the cyst wall with leakage of fluid and surrounding inflammation may present with pain in 20% cases.[6] The differential diagnosis includes lipoma, epidermoid cysts etc, hence the diagnosis was missed in our cases.

High-resolution sonography can clinch the diagnosis by demonstrating presence of a scolex within the cyst. It was not done in any of our patients as the diagnosis of subcutaneous cysticercosis was not suspected. Other sonographic features described are surrounding oedema or abscess formation, which are often seen after death of the larvae.[3,7,8] Rarely, multiple calcified cysticerci in the soft tissues may be seen even on plain radiograph as rice grain appearance.[8] Isolated soft tissue cysticercosis is often used as a marker of neurocysticercosis and an evaluation for co-existing central nervous system and ocular involvement is recommended.[6] This was done postoperatively in our patients.

Fine needle aspiration cytology is extremely useful in reaching a preoperative diagnosis of soft tissue cysticercosis. The aspirate is usually blood stained. Clear fluid and a pearly white membrane are seen in 14.4% and 6% of cases respectively.[9] The presence of tiny parasitic fragments like spiral larval walls and detached hooklets in an inflammatory background of eosinophils and histiocytes can diagnose cysticercosis with certainty.[9] In a study of 132 patients, Khurana et al demonstrated parasitic fragments on FNAC in 75% cases.[9] In our patients FNAC was done only in 2 patients, none of which could confirm the diagnosis.

Surgical excision of isolated soft tissue cysticercosis usually suffices if concurrent involvement of central nervous system and ocular disease have been ruled out. Conversely, if a definite diagnosis has been made on the basis of ultrasonography and FNAC without any evidence of abscess formation, surgery may not be required at all.[3] Complete resolution of the cysticercosis has been shown with high dose antihelminthic therapy with either praziquantel (50-75 mg/kg per day for 15-30 days), or albendazole (10-15 mg/kg per day for 8 days).[3,10]

In endemic areas like ours, isolated soft tissue cysticercosis must be considered as a differential diagnosis for subcutaneous nodules on the trunk. If diagnosed accurately, it can be treated medically, eliminating the need for surgery.

## Footnotes

**Source of Support:** Nil

**Conflict of Interest:** None declared

